# Does COVID-2019 Have an Impact on the Purchase Intention of Commercial Long-Term Care Insurance among the Elderly in China?

**DOI:** 10.3390/healthcare8020126

**Published:** 2020-05-06

**Authors:** Xiaocang Xu, Lu Zhang, Linhong Chen, Feng Wei

**Affiliations:** 1 School of Economics, Chongqing Technology and Business University, Chongqing 400067, China; cangxiaoxu@ctbu.edu.cn; 2 Research Center for Economy of Upper Reaches of the Yangtse River, Chongqing Technology and Business University, Chongqing 400067, China; melodyzl@163.com; 3 School of Mathematics and Statistics, Chongqing Technology and Business University, Chongqing 400067, China; 2017325010005@stu.scu.edu.cn; 4 School of Public Administration, Sichuan University, Chengdu 610065, China; 5 School of Management and Economics, University of Electronic Science and Technology of China, Chengdu 611731, China

**Keywords:** COVID-2019, commercial long-term care insurance (LTCI), Andersen behavioral model, long-term care (LTC), disabled elderly

## Abstract

Purpose: As an important measure to alleviate long-term care (LTC) costs for the disabled due to the aging of the population, long-term care insurance (LTCI) system has been paid more attention in China. In addition to the government-led public LTCI system that has been piloted in cities such as Qingdao, Chongqing and Shanghai, health insurers such as the China Life Insurance Company are also experimenting with various types of commercial LTCI in the private market. However, the commercial LTCI market is developing very slowly due to public awareness and other reasons. On the other hand, COVID-2019 has had an impact on the cognition of the importance of long-term care for the elderly due to the fact that the death cases of COVID-2019 have been mainly concentrated in the elderly population with chronic diseases such as hypertension. Therefore, the purpose of this study is to explore the differences in the purchase intention of commercial LTCI among the elderly in two different periods: before and after the outbreak of COVID-2019. Methods: By using the Andersen behavioral model and two investigations in two different periods before and after the outbreak of COVID-2019, this study explores the impacts of COVID-2019 on the purchase intention of commercial LTCI. Results: Some significant discoveries were found. For example, 25.8% of interviewees showed purchase intention in LTCI in the time before the COVID-2019 outbreak, while this proportion increased to 37.6% after the COVID-2019 outbreak. People who were younger (OR = 2.128, before COVID-2019; OR = 1.875, after COVID-2019) or who had more education (OR = 1.502, before COVID-2019; OR = 2.218, after COVID-2019) were more interested in commercial LTCI. Conclusion: This study shows that COVID-2019 has had an obvious impact on the purchase intention of commercial LTCI, which provides some enlightenment for China to improve the LTCI system in the future, especially to accelerate the development of commercial LTCI. For example, it is essential to promote the importance of long-term care among the elderly in a focused and targeted way. In terms of the key target audience, it can be developed gradually from the groups with higher education levels and the middle elderly aged 45–64 years old.

## 1. Introduction

The aging population in some countries has led to a growing number of disabled elderly, creating a massive need for long-term care and increased costs, which is a heavy economic burden on the families of the disabled elderly and governments. For example, aging of the population has created an immense demand for long-term care (LTC) with a dramatic increase in chronic diseases in China. In 2016, the total disability rate of the elderly in China was 16.5%, of which the severe disability rate was 7.2%. According to projections, LTC costs for the disabled elderly will increase from US $37.82 billion in 2016 to US $68.69 billion in 2020 and US $246.76 billion in 2050 [1]. Long-term care (LTC) refers to “a system of care activities carried out by informal caregivers (family, friends or neighbors) and professionals (health and social services) to ensure that those who do not have full self-care capacity continue to enjoy a higher quality of life” (WHO) [2]. Long-term care (LTC) service forms can be either home care, i.e., professional nursing personnel door-to-door service or institutional care, i.e., including in nursing homes, apartments for the elderly and other non-hospital professional nursing institutions for professional care. In 2016, there were 220 million people over the age of 60 and nearly 10 million completely disabled elderly that could not manage activities of daily living (ADLs). Of these, only 18.5 percent benefit from basic health insurance and public long-term care services, which are being piloted in 15 cities [3]. How to meet the needs of LTC and establish a sustainable LTC system is becoming a long-term and severe worth consideration policy problem [4,5,6,7,8,9]. The lack of adequate understanding of LTC demand and supply leads to inadequate financial preparation and payment difficulties for the elderly [10]. For many older people and their families, out-of-pocket payments for LTC represent a catastrophic economic burden. In this context, LTCI emerged to solve this problem.

### 1.1. The Development of LTCI System

LTCI (long-term care insurance), including public LTCI (government-mandated) and commercial LTCI (private voluntary purchase), refers to an institutional arrangement that focuses on providing nursing protection and financial compensation for the insured in case of loss of daily living ability, old age, illness or death [11]. Taking China as an example, the public LTCI refers to the long-term care fund pool, which is made up of a certain proportion of funds from the basic medical insurance of individuals, and it has the characteristics of mandatory, wide coverage, but few long-term care services. commercial LTCI is a long-term care insurance variety developed by commercial insurance companies, such as CPIC (Pacific Insurance Agency), for individuals and families. It has the characteristics of voluntary, high cost, but many long-term care services.

Public LTCI systems have been implemented in Germany and other European countries since the 1990s, which has alleviated the crisis of LTC services cost for the disabled elderly. In China, healthcare is unbalanced or inadequate because of the aging population, and there is a severe contradiction between them and the health demands of the people [12]. A dramatic increase in the elderly population highlights the crisis of healthcare for the elderly. The elderly above 65 increased from 96.2 million in 2003 to nearly 150.8 million in 2016, with many of them disabled due to chronic diseases. Hence, the need for LTC services is snowballing, but its fund supply lags far behind. Since 2012, the Chinese government has pushed a sequence of policies or measures to build a thorough healthcare system for the disability elderly such as the pilot of LTC insurance (LTCI) [13,14,15]. Some cities such as Qingdao, Shanghai, and Chongqing have already taken LTCI as a critical task from 2016 to 2019. Professional insurance is considered a utility to meet LTC costs because it regularly pays predictable and affordable premiums, thereby reducing the risk of catastrophic out-of-pocket costs. The work of the private sector may alleviate the growing economic cost of public LTC spending or self-pay payment.

Commercial LTCI in the private market is not enough development and contributes very little to the financial markets and long-term care for the elderly [16]. Despite its potential, commercial LTCI has inherent weaknesses that prevent it from expanding into the long-term care market, such as its too high price [17,18] and the risk due to severe information asymmetry [19]. Thus, the commercial LTCI market has far failed to flourish even in an aging society with a liberal economy. For instance, only 1%–2% of LTC spending was funded by commercial LTCI in most OECD countries [20]. Some scholars believed that the main reason for the underdevelopment of the commercial LTCI market lies in the supply factors [21]. Other studies, on the contrary, have revealed that the demand factors, such as the absence of knowledge of future LTC risks, intra-family moral hazard and other factors, will continue to impede the development of the commercial LTCI market [22,23,24,25,26,27,28]. A recent study revealed that the needs of commercial LTCI of the elderly in America were affected by the following elements: preferences, substitutes for LTCI, such as public welfare and characteristics of insurance [29]. Other studies have illustrated the crowding-out effect of informal care [30] in demand for commercial LTCI. Gender is another feature that may impact the demand for LTCI. As women have a longer life expectancy, it was estimated that they have a higher average number of remaining disability years than men. As a result, older women are also more likely to use formal LTC services than men led to the exists of gender heterogeneity in the interest to purchase LTCI [31]. Therefore, the analysis of gender differences is also one of the contents of this study.

### 1.2. COVID-2019 and Long-Term Care for the Elderly

The COVID-2019 outbreak of December 2019 continues to spread around the world. The COVID-2019 crisis has attracted much attention because of its high harmfulness. The impact of COVID-2019 on th mental health of medical staff and patients have been the focus of many studies [32,33], which has also begun to draw attention to long-term care for the elderly [34,35,36,37].

**Figure 1:**
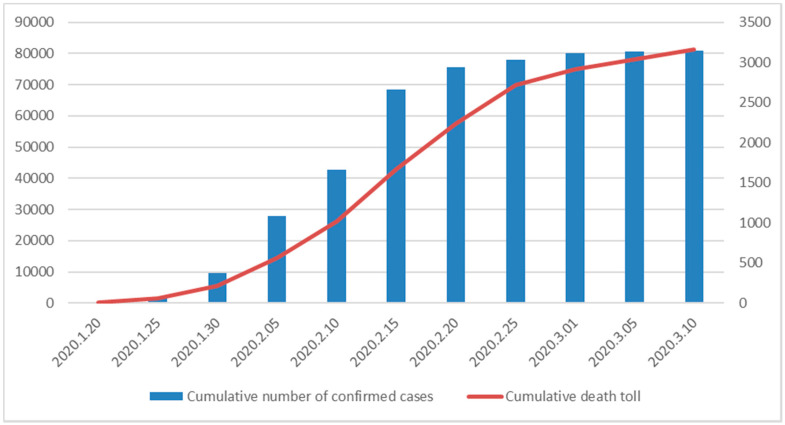
The trend of COVID-2019 in the Hubei province of China.

According to the National Health Commission of the People’s Republic of China, as of April 4 2020, 1,134,775 patients have been diagnosed, and 62,448 patients have died around the world. Most of the deaths were among the elderly with chronic diseases such as high blood pressure, diabetes and chronic bronchitis. For example, the median age of death was 72 years, and the proportion of deaths among the elderly was significantly higher than the percentage of other infections in the Hubei province of China (Figure 1). As can be seen from the above, incapacitated or semi-incapacitated the elderly suffering from chronic diseases with weak immunity have become the primary victims of COVID-2019. The fact that the high post-cure care cost of COVID-2019 and the aging of its death cases will certainly prompt people, especially the elderly, to have a new understanding of LTC (Long term care) and LTCI (long term care insurance), and the purchase intention of commercial LTCI will certainly change.

To sum up, commercial LTCI’s purchase intention is influenced by many factors, including cultural background and external environment [38,39,40,41,42,43,44]. In China, the general public knows little about the LTCI (long term care insurance) system or is consciously avoiding commercial LTCI. The COVID-2019 shock may have an impact on this, raising awareness of the importance of long-term care for the elderly and increasing interest in LTCI purchases. Therefore, this study tries to discuss the influence of COVID-2019 on the buying intentions of commercial long-term care insurance in the periods before and after the outbreak of COVID-2019. The structure of this study is as follows: the questionnaire process and model framework were shown in the second chapter, and the third chapter was the results of multivariate logistic regression. Finally, the discussion and conclusion were, respectively in chapters 4 and 5.

## 2. Materials and Methods

### 2.1. Questionnaire Process and Sample

In order to compare the differences in the buying intentions of commercial long-term care insurance among the elderly in the two different periods before and after the outbreak of COVID-2019, this study used the results of two questionnaires.

The first questionnaire took place before the outbreak of COVID-2019. It performed by Sichuan University and Chongqing Technology and Business University from July 15 to September 1, 2019 (that is, during the summer vacation of Chinese universities), consisted of 15 questions (only 12 variables were included in the later empirical analysis, and other variables were not included due to too many missing values, Table A1). The questionnaire was conducted in among elderly living in urban communities (divided into two groups: aged 45–64 and over 65) in four representative cities (Guangzhou, Shanghai, Chongqing and Chengdu), which was based on the following reasons: commercial long term care insurance is a supplement to the government’s public long term care insurance and medical insurance, and only the groups with higher incomes or living in higher economic development levels regions have the interesting in commercial long term care insurance. The completion time of the interviewees was about 8–15 minutes and answered the questions anonymously. A total of 3584 samples were recovered (with the help of the district commissioners of the various urban communities) and 2772 valid samples were collected (The effective rate was 77.3%). There were three main criteria for sample exclusion: a) samples with missing values, b) samples under the age of 45 years, and c) if the subject claims to not have a basic understanding of LTCI, even after a detailed introduction (We had a detailed introduction of LTCI before the formal question, followed by the question: do you have a basic understanding of LTCI? If the answer was NO, we removed this sample before asking further questions. Of course, before the formal questions in the survey questionnaire, we made a brief introduction to the concept of commercial LTCI, which is described in detail in the next section). It should be noted that we were writing and improving the analysis of the results of this questionnaire since October 2019, but it has not been published yet. The COVID-2019 outbreak in December 2019 in the process of writing that study, so we decided to conduct a second questionnaire survey again to discuss the impact of COVID-2019.

The second questionnaire took place after the outbreak of COVID-2019 from January 25th to March 10th, 2020. The second questionnaire was conducted in the same communities as the first field survey. We found some partners, such as the local community managers or village cadres, in the area of our first field survey to help us and tell them the purpose and difference of the second survey. Then, we ask them to issue online questionnaires, made by a popular network research software ‘Questionnaire Star (Chinese: ***问卷星***)’, in their community or village by WeChat group and QQ group with prizes answer. And tell them in advance that if they complete the research questions carefully, they will get a small gift such as laundry night or meal study. The difference between them and the field research may have an impact on the results: when the respondents have any questions or don’t understand the content, the field research can be explained face to face, which is more beneficial than the online survey. Therefore, in order to overcome this problem, LTCI was introduced in more detail at the beginning of the questionnaire when we designed the new questionnaire. COVID-2019, highly infectious, leading to the current travel control in all regions of China. Therefore, the traditional way of issuing questionnaires is no longer suitable. As a new tool, the Internet has been closely connected with the Chinese people and used more and more in psychological research with increasingly mature technology. We adopted this method in our research because of its advantages, such as fast release and short cycle. At the same time, we also optimized the question design to overcome the shortcomings of the network survey itself to the greatest extent, such as adding a few polygraphs and common sense questions to identify the interviewees who did not answer carefully, to ensure the reliability of the answers. All the questionnaire results were directly transferred to R software for further data processing and statistical analysis. In addition, at the end of the sample collection, people under the age of 45 or samples with missing values were excluded. We used the same questions as the first questionnaire, but only collected a total of 1428 samples were recovered and 1215 valid samples (The effective rate was 85.1%).

### 2.2. Model Framework

To consider various factors that may affect the interest in commercial LTCI, the Andersen Behavioral Model was used as our model framework [45]. The model has included three groups of factors: a) predisposing factors, such as gender or age that may influence behavior related to long-term care even before the onset of the disease, b) enabling factors that facilitate or limit the use of services in the event of illness, such as family support, insurance, etc. and, c) need factors that highlight perceived and actual nursing needs and have a direct impact on service utilization. In this study, the Andersen model—widely used in empirical research to analyze the demand of LTCI—was used to explain the factors affecting the interest of individuals to buy commercial LTCI in China.

### 2.3. The Dependent Variable

In reference to related literature [46,47,48,49,50], the dependent variable was the purchase intention of commercial LTCI in interviewees. The tool includes questions about measuring personal social background, income situation, awareness for LTCI and expected LTC needs. Note that the LTCI in this study was independent of general individual health or life insurance. In detail, with reference to the study of He, A. J and Chou, K. L [51], the questions are designed as follows: long-term-care insurance, separated from your regular health insurance, is an insurance policy that helps families of the disabled elderly pay for personal Care to relieve economic stress. Would you be interested in an insurance product that requires you to pay regular premiums (about 7068 yuan/year) for five years, but provides you with economic protection against the economic risk of long-term care costs (1000 yuan per month until the long-term care status is discontinued)? It has two choice answers: Yes or No, which assigned 1 and 0, respectively.

### 2.4. The Independent Variables

#### 2.4.1. Predisposing Factors

Predisposing factors mainly included aged 45–64 and over 65 two groups, gender, living state (for example living with offspring or not; how many children), marriage status, education level and the following two attitudinal statements regarding LTCI (referred to in [23,46]): (a) availability of family long-term care (Question: “Will your family member take care of you if you need long-term care?” Answer: 1 = no, 2 = yes); and (b) burden on family financial when need long-term care (Question: “Do you feel there is an economic burden on your family when you need long-term care?” Answer: 1 = no, 2 = yes).

#### 2.4.2. Enabling Factors

Enabling factors mainly included the following items: availability of savings and the ability to pay for LTCI. (1) the availability of savings (money left over from normal expenditure planning) was obtained by asking, “Do you have any extra money at the end of each month for discretionary income?”. Because we believe that people, in most cases, are only interested in buying long-term-care insurance once they have met the basic cost of life planning. (2) The ability to pay for LTCI was referred to the study by Brown et al. [23] and He, A. J & Chou, K. L [51]: (a) pay for LTC cost by government welfare; (b) the ability to pay LTCI premiums. They were told about the approximate pricing level of LTCI offered by CPIC (Pacific Insurance Agency) before answering those questions.

#### 2.4.3. Needs Factors

Needs factors contained the perceived possibility of LTC needs and expected dependence in the future [52]. The way to assess LTC needs is to ask interviewees, “What is your evaluation of your opportunities to the LTC need (including home care and formal care such as institutional care) in the future?” The expectation of dependencies was observed by asking interviewees to what extent do they agree with this statement, “Sometime in the future, it is likely that you will need help to live due to health problems” on five scales. All variables were split into two groups.

### 2.5. Ethical Considerations

This study did not involve human clinical trials and the questionnaires were anonymous, so it does not need to provide ethical review documents or informed consent. Participants of the study were informed about their right to withdraw from the study at any time. Finally, informed written consent was obtained before participation, and confidentiality of the information was maintained by omitting personal identifiers.

### 2.6. Empirical Methods and Procedures

The logistic regression analysis process was performed in three steps. First, we carried out and compared bivariate statistical analysis according to predisposing, enabling and needs factors between two questionnaires. Second, a comparison of multivariate analysis was made to explore the impact of COVID-2019 on the purchase intention of commercial LTCI among the Elderly in China. At the end of the empirical analysis, we made two further comparisons of sub-analyses, namely, by gender and by Educational level.

## 3. Results

### 3.1. Descriptive Statistics and Bivariate Analysis

**Figure 2:**
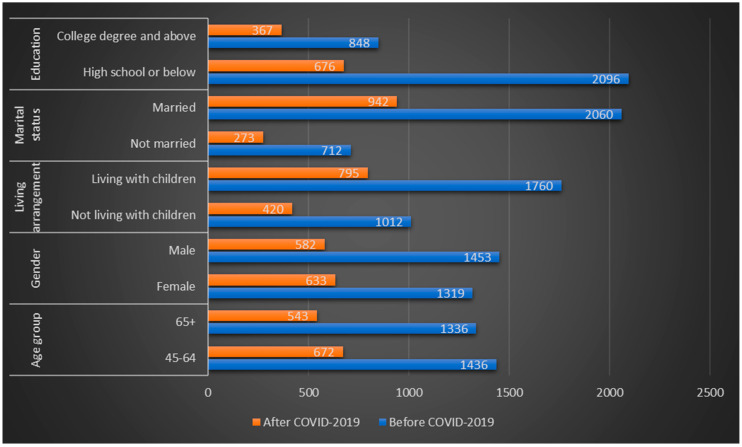
Sample characteristics of the two questionnaires.

The purpose of this study was to discuss the influence of COVID-2019 on the buying intentions of commercial long-term care insurance in the periods before and after the outbreak of COVID-2019. Therefore, descriptive statistics results of all variables in the two periods were shown in Figure 2 and Table 1.

As shown in Figure 2, the reason why the sample size of the second questionnaire was significantly reduced may be related to two reasons. First, many people go to other places for family reunion or travel during the Chinese New Year. Second, it is affected by the COVID-2019 outbreak. However, as can be seen from Figure 2 and Table 1, there is little difference in the basic characteristics between the two questionnaires. Therefore, the potential influence of the different sample sizes will definitely exist, but not too large.

Overall, 25.8% of interviewees showed purchase intention in LTCI in the time before the COVID-2019 outbreak, while this proportion increased to 37.6% after the COVID-2019 outbreak.

For predisposing factors, among all the interviewees in the time before COVID-2019 outbreak, 48.2% (44.7%, after COVID-2019 outbreak) were the elderly over 65, 52.4% (47.9%, after COVID-2019 outbreak) were male, 63.5%(65.4%, after COVID-2019 outbreak) live with their children, 74.3% (77.5%, after COVID-2019 outbreak) were in married and 24.4% (30.2%, after COVID-2019 outbreak) had a bachelor’s degree or above. More than 67.3% (65.3%, after COVID-2019 outbreak) self-confirmed the availability of home care when they need long-term care; 69.6% (72.2%, after COVID-2019 outbreak) said that their LTC needs were an economic burden to some extent on their families.

For enabling factors, 67.2% (69.5%, after COVID-2019 outbreak) said they often had money left over regularly for extra expenses; 19.8% (16.4%, after COVID-2019 outbreak) said that their LTC spending was covered by welfare; 60.8% (61.9%, after COVID-2019 outbreak) reported that LTCI premiums were affordable.

For needs factors, 27.2% (44.4%, after COVID-2019 outbreak) predicted to need LTC and 22.7% (21.8%, after COVID-2019 outbreak) of these predicted to have the dependent on LTC in the future.

In addition, as revealed in Table 1. Bivariate analysis showed a statistically significant correlation between the purchase intention of commercial LTCI and gender, living arrangement, education and the economic burden on the family in the two periods.

### 3.2. Multivariate Analyses Results

Table 2 displayed the comparison of the multivariate logistic regression results related to the purchase intention of commercial LTCI in two periods. Chi-squared significance statistics showed that predictors of dependent variables of each factor subset were reliable, and the explained variance percentage was moderate.

In the period before COVID-2019, the elderly aged 45–64 show stronger purchase intention than the elderly aged over 65 (OR = 2.218). Males showed stronger purchase intention of commercial LTCI than females (OR = 1.326). People with college education or above show stronger purchase intention than people without college education (OR = 1.502) and those who think they have the ability to pay the insurance expenses show stronger purchase intention than those without (OR = 1.711). In addition, the two variables in need factors show apparent differences.

In the period after COVID-2019, the elderly aged 45–64 show stronger purchase intention of commercial LTCI than the elderly aged over 65 (OR = 1.875). People with college education or above show stronger purchase intention than people without college education (OR = 2.218), and those who think they have the ability to pay the insurance expenses show stronger purchase intention than those who without (OR = 1.379).

In terms of comparing the two time periods, most of the factors, such as age and education, were similar in terms of purchase intentions. However, on the whole, the differences among different groups such as age and gender were narrowing after COVID-2019 except education factors. People with college education or above show stronger purchase intention than people without college education (OR = 2.218) after COVID-2019 outbreak compares to before COVID-2019 (OR = 1.502).

Considering the relationship with the family, the elderly with the ability to obtain home care have a lower purchase intention for commercial LTCI, compared with those without the ability to obtain home care. Purchase intention improved but not by much after COVID-2019. The same changes were made in the elderly whether or not to have some welfare to pay LTC. However, the elderly who believed that LTC would impose a certain economic burden on their families had a stronger intention to purchase commercial LTCI after COVID-2019 (OR = 0.918) than before COVID-2019 (OR = 0.661).

Moreover, the elderly who need LTC more than those think they do not need LTC have stronger purchase intention to commercial LTCI (OR = 1.455) before COVID-2019, but this gap has narrowed (OR = 1.107) after COVID-2019. This may not be because the purchase intention of the elderly who need LTC was reduced, but because people who think they do not need LTC switched to commercial LTCI purchase intention stronger due to the effect of COVID-2019. The same changes occurred in the elderly who had different perceptions of whether they needed help with their health or not (OR = 1.621, before COVID-2019 outbreak; OR = 1.285, after COVID-2019 outbreak).

### 3.3. Sub-Multivariate Analysis by Gender

It can be seen from the above that factors such as gender and education level have a great influence on the purchase intention of commercial LTCI. Therefore, we carried out sub-analyses by gender and by educational level next.

Some significant results can be seen in Table 3.

First, some factors, such as living arrangements and marital status, were not sensitive to the impact of COVID-2019. For example, men who live with children showed a more clear purchase intention of commercial LTCI than females, regardless of the period.

Second, from the perspective of age, both men and women aged 45–64 showed significantly stronger purchase intention of commercial LTCI than those aged over 65 in the two time periods before and after COVID-2019 outbreak. However, men were more sensitive to the impact of COVID-2019. This characteristic also has the same embodiment in the educational factor.

### 3.4. Sub-Multivariate Analysis by Educational Level

Table 4 displayed the comparison of the multivariate logistic regression results by educational level related to the purchase intention of commercial LTCI in two periods.

First, in the period before COVID-2019, people with a college degree and above had a stronger interest in buying commercial LTCI than those with high school or below, which was also consistent with the general perception of our society. However, surprisingly, the performance of “anticipation of being dependent” in different education levels presents certain particularity. The performance of people with a college degree and above (OR = 2.333) was even more outstanding than those with high school or below (OR = 1.822).

Second, some factors, such as marital status and availability of savings, were not sensitive to the impact of COVID-2019. For example, people with a college degree and above showed little diffidence in purchase intention of commercial LTCI than those with high school or below in two periods regardless of married or not.

Third, a few factors like age and living arrangements were more affected by COVID-2019. For example, college-educated people who live with their children after COVID-2019 showed more definite purchase intention of commercial LTCI than those before COVID-2019.

## 4. Discussion

By using the Andersen behavioral model, this study attempts to answer two questions: (a) Which factors have a great influence on the purchase intention of commercial LTCI (long-term care insurance) among the elderly in China? (b) Does COVID-2019 have an obvious impact on the purchase intention of commercial LTCI in China?

First, 25.8% of interviewees showed interest in having a commercial LTCI plan in the period before COVID-2019 and this proportion increases to 37.6% in the period after COVID-2019, which reveals that COVID-2019 has had an obvious impact on the purchase intention of commercial LTCI in China. Even considering some possible error, these data still give an index to a rather inspiring message and has positive implications for LTCI policy. In America, where commercial LTCI coverage was the strongest, only 5% among the adults aged 40 and over have a commercial LTCI planning [20].

Second, the typical feature of the elderly in China who likely to buy LTCI in the future included: Younger (OR = 2.128, before COVID-2019; OR = 1.875, after COVID-2019), received more education (OR = 1.502, before COVID-2019; OR = 2.218, after COVID-2019), more ability to pay the premium (OR = 1.711, before COVID-2019; OR = 1.379, after COVID-2019), etc. These characteristics were evident both before and after the COVID-2019 outbreak. It can also be revealed that the impact of COVID-2019 on the purchase intention of commercial LTCI was mainly reflected in different educational groups. In addition, the elderly who live with children may be less urgent to fund their Long-term care services by commercial LTCI and less interested in commercial LTCI [51]. However, COVID-2019 had some impact on this idea, as they are concerned that the high treatment costs of a disaster such as COVID-2019 in the future will constitute a financial burden on the children’s families.

Third, although only 19.8% (before COVID-2019; 16.4%, after COVID-2019) of interviewees said that public assistance would pay for their LTC cost, this seemed to have nothing to be related to the interest to buy commercial LTCI in China. It can be explained as interviewees believe that perceived benefits are the last one of the resorts and do not know the status of LTC financing. These discoveries echo some previous researches in the Western developed countries [31,46]: The existence of government public LTCI or health insurance could significantly crowd out the individual plans about commercial LTCI [53,54]. Generous public LTC subsidies have had much the same effect, by providing poor old people with a comfortable last resort. In addition, the elderly who believed that LTC would impose a certain economic burden on their families had a stronger intention to purchase commercial LTCI after COVID-2019 (OR = 0.918) than before COVID-2019 (OR = 0.661). It also potentially illustrates that household income is also an important factor in the purchase intention of commercial LTCI. The elderly who need LTC than those think themself do not need LTC have stronger purchase intention to commercial LTCI (OR = 1.455) before COVID-2019, but this gap has narrowed (OR = 1.107) after COVID-2019. It may due to the increase of the purchase intention for commercial LTCI among the elderly who think themself do not need LTC. All these phenomena show that COVID-2019 has a great impact on the purchase intention of commercial LTCI.

In the end, sub-multivariate analysis by gender and by educational level was carried out, which took all three factors, namely, the predisposing factors, the enabling factors and the need factors into consideration. It is found that male has more interest to buy commercial LTCI than female on the whole in China. It may be due to the differences between men and women in the structure of the social division of labor and the economic responsibilities of the family. It has some similarities with the conclusion in the study of Zeng, et al [36]. In addition, in the period of before COVID-2019, people with a college degree and above have a stronger interest in buying commercial LTCI than those with High school or below, which is also consistent with the general perception of our society. However, surprisingly, the performance of “Anticipation of being dependent” in different education levels presents certain particularity. The performance of people with a college degree and above (OR = 2.333) was even more outstanding than those with High school or below (OR = 1.822).

In conclusion, this study shows that COVID-2019 has an obvious impact on the purchase intention of commercial LTCI, which provides some enlightenment for China to improve the LTCI system in the future, especially to accelerate the development of commercial LTCI. First of all, it is very important to promote the importance of long-term care among the elderly in a focused and targeted way. In terms of the key target audience, it can be developed gradually from the groups with higher education levels and the middle elderly aged 45–64 years old. Second, the promotion of the LTCI system could be strengthened in the light of the heavy financial burden on households resulting from estimates of the costs of the disaster in China. Third, the Chinese government can try to effectively link commercial LTCI and public LTCI to complement each other and effectively solve the gap between the supply and demand of long-term care services caused by the rapid growth of the aging population.

## 5. Conclusions

Through the comparison between the two surveys of before and after COVID-2019, this study finds that COVID-2019 has an obvious impact on the purchase intention of commercial LTCI, which is mainly reflected in the factors such as education level, the ability to pay the premium, household income and the elderly’s self-perception of their own health status. The reason should be related to the high post-cure care cost of COVID-2019 and the aging of death cases. Therefore, it is important to publicize the harm caused by sudden public health events like COVID-2019 to make the general public more aware. Governments should carry out more education and publicity work aimed at the middle and older age groups and the more educated groups to raise their awareness of the cost of LTC and their interest in purchasing commercial LTCI. Meanwhile, in order to avoid selection problems in commercial LTCI as much as possible, the government should improve the public LTCI system as much as possible and extend the implementation scope of public LTCI system from 15 pilot cities to other cities as soon as possible. There are some limitations in this study, for example, the sample size of the two questionnaires before and after COVID-2019 was not equal, as well as other factors that can reflect the psychological changes of the interviewees, we will make further research in the future.

## Figures and Tables

**Table 1:** Comparison of bivariate analyses of the effects of predisposing, enabling and needs factors on the purchase intention of commercial long-term care insurance (LTCI) in the period of before and after COVID-2019.

Factors	Before COVID-2019 (*n* = 2772)			After COVID-2019 (*n* = 1215)		
	Proportion %	Purchase Intention %	χ2	Proportion %	Purchase Intention %	χ2
Total sample		25.8			37.6	
1.Predisposing factors						
(1) Age group			53.25 **			47.33 **
45–64	51.8	35.2		55.3	44.4	
65+	48.2	17.3		44.7	21.6	
(2) Gender			7.36 ***			7.92 ***
Female	47.6	37.5		52.1	46.8	
Male	52.4	39.6		47.9	49.5	
(3) Living arrangement			27.53 ***			23.21 **
Not living with children	36.5	38.7		34.6	48.3	
Living with children	63.5	28.6		65.4	35.7	
(4) Marital status			5.88 *			6.18 *
Not married	25.7	37.2		22.5	46.5	
Married	74.3	25.3		77.5	31.6	
(5) Education			19.25 ***			17.78 ***
High school or below	75.6	27.8		69.8	34.7	
College degree and above	24.4	43.2		30.2	54.2	
(6) Availability of home care			3.55 *			3.91 *
No	32.7	36.5		34.7	45.6	
Yes	67.3	23.2		65.3	29.8	
(7) Burden on family			47.28 ***			50.12 ***
No	30.4	11.2		27.8	14.4	
Yes	69.6	30.3		72.2	37.8	
2.Enabling factors						
(8) Availability of savings			36.88			31.25
No	32.8	15.5		30.5	19.3	
Yes	67.2	38.2		69.5	47.7	
(9) Paying LTC by welfare			0.19 *			0.17 **
No	80.2	31.8		83.6	39.5	
Yes	19.8	15.8		16.4	19.7	
(10) Ability to afford premium			0.04			0.03
No	39.2	22.3		38.1	27.8	
Yes	60.8	41.2		61.9	51.5	
3.Needs factors						
(11) Likelihood of needing LTC			8.65 **			9.07 **
No	72.8	19.2		55.6	24.2	
Yes	27.2	44.0		44.4	55.1	
(12) Anticipation of being dependent			14.21 *			13.18 *
No	77.3	22.6		78.2	28.2	
Yes	22.7	42.5		21.8	53.1	

Note. * *p* < 0.1, ** *p* < 0.05, *** *p* < 0.01.

**Table 2:** Comparison of multivariate analyses of the effects of predisposing, enabling and needs factors on the purchase intention of commercial LTCI in the period before and after COVID-2019.

Factors	Before COVID-2019 (*n* = 2772)			After COVID-2019 (*n* = 1215)		
	OR	95% CI		OR	95% CI	
		Lower Limit	Upper Limit		Lower Limit	Upper Limit
1.Predisposing factors						
(1) Age group						
65+ (ref.)						
45–64	2.128 **	1.745	2.511	1.875 **	1.518	2.184
(2) Gender						
Female (ref.)						
Male	1.326 ***	1.168	1.484	1.118 ***	1.016	1.291
(3) Living arrangement						
Not living with children (ref.)						
Living with children	0.312 *	0.071	0.553	0.355 **	0.161	0.481
(4) Marital status						
Not married (ref.)						
Married	0.913	0.840	0.986	1.133	0.731	1.457
(5) Education						
High school or below (ref.)						
College degree and above	1.502 ***	1.241	1.764	2.218 ***	1.979	2.534
(6) Availability of home care						
No (ref.)						
Yes	0.795 *	0.654	0.936	0.877 *	0.668	0.974
(7) Burden on family						
No (ref.)						
Yes	0.661 **	0.404	0.918	0.912 ***	0.739	1.249
2.Enabling factors						
(8) Availability of savings						
No (ref.)						
Yes	1.502 **	1.286	1.718	1.398 *	1.118	1.494
(9) Paying LTC by welfare						
No (ref.)						
Yes	0.709 *	0.529	0.889	0.828 ***	0.569	0.973
(10) Ability to afford premium						
No (ref.)						
Yes	1.711 **	1.462	2.260	1.379 **	0.836	1.796
3.Needs factors						
(11) Likelihood of needing LTC						
No (ref.)						
Yes	1.455 *	1.304	1.606	1.107 *	0.934	1.597
(12) Anticipation of being dependent						
No (ref.)						
Yes	1.621 **	1.401	1.841	1.285 **	1.018	1.801
Constant	0.019			0.022		
Model summary chi square	267.16			255.35		
(df, *p* value)	(13, *p* <.001)			(13, *p* <.001)		
–2 Log likelihood	1708.56			1554.68		
Nagelkerke R2	0.28			0.27		

Note. OR = odds ratio; * *p* < 0.1, ** *p* < 0.05, *** *p* < 0.01.

**Table 3:** Sub-multivariate analysis by gender.

Factors	Before COVID-2019 (*n* = 2772)		After COVID-2019 (*n* = 1215)	
	Male	Female	Male	Female
	OR	OR	OR	OR
Predisposing factors				
(1) Age group				
65+ (ref.)				
45–64	2.825 ** (0.076)	2.213 ** (0.102)	3.208 ** (0.058)	2.556 ** (0.097)
(3) Living arrangement				
Not living with children (ref.)				
Living with children	0.443 *** (0.111)	0.324 *** (0.098)	0.425 *** (0.127)	0.312 *** (0.094)
(4) Marital status				
Not married (ref.)				
Married	0.887 (0.107)	0.732 (0.211)	0.855 * (0.153)	0.745 * (0.208)
(5) Education				
High school or below (ref.)				
College degree and above	1.806 *** (0.364)	1.313 *** (0.277)	2.479 *** (0.275)	2.308 *** (0.232)
(6) Availability of home care				
No (ref.)				
Yes	0.701 (0.155)	0.834 (0.141)	0.522 * (0.123)	0.635 * (0.108)
(7) Burden on family				
No (ref.)				
Yes	1.898 *** (0.608)	3.632 *** (0.523)	1.155 *** (0.575)	2.532 *** (0.444)
2.Enabling factors				
(8) Availability of savings				
No (ref.)				
Yes	1.428 * (0.213)	1.513 * (0.234)	1.125 * (0.135)	1.476 * (0.093)
(9) Paying LTC by welfare				
No (ref.)				
Yes	0.718 (0.137)	0.635 (0.176)	0.776 ** (0.125)	0.708 ** (0.108)
(10) Ability to afford premium				
No (ref.)				
Yes	1.545 ** (0.134)	1.135 ** (0.166)	1.266 * (0.113)	1.089 * (0.116)
3.Needs factors				
(11) Likelihood of needing LTC				
No (ref.)				
Yes	1.985 (0.168)	1.378 (0.112)	2.187 (0.108)	1.754 (0.098)
(12) Anticipation of being dependent				
No (ref.)				
Yes	1.922 * (0.216)	1.477 * (0.189)	1.821 * (0.208)	1.582 * (0.122)
Constant	0.039	0.031	0.027	0.023

Note. OR = odds ratio; * *p* < 0.1, ** *p* < 0.05, *** *p* < 0.01. SD value is in parentheses.

**Table 4:** Sub-multivariate analysis by educational level.

Factors	Before COVID-2019 (*n* =2772)		After COVID-2019 (*n* = 1215)	
	High School or Below	College Degree and Above	High School or Below	College Degree and Above
	OR	OR	OR	OR
Predisposing factors				
(1) Age group				
65+ (ref.)				
45–64	1.872 * (0.334)	2.266 ** (0.256)	2.579 ** (0.254)	2.822 ** (0.208)
(2) Gender				
Female (ref.)				
Male	1.521 * (0.154)	1.625 * (0.121)	1.478 *** (0.104)	1.558 *** (0.111)
(3) Living arrangement				
Not living with children (ref.)				
Living with children	0.289 ** (0.243)	0.457 ** (0.109)	0.886 ** (0.202)	0.565 ** (0.158)
(4) Marital status				
Not married (ref.)				
Married	0.715 (0.188)	0.924 (0.178)	0.923 * (0.185)	0.755 * (0.108)
(6) Availability of home care				
No (ref.)				
Yes	0.723 (0.125)	0.815 (0.112)	0.915 (0.137)	0.788 (0.098)
(7) Burden on family				
No (ref.)				
Yes	3.112 *** (0.619)	2.578 *** (0.598)	3.275 *** (0.517)	3.556 *** (0.622)
2.Enabling factors				
(8) Availability of savings				
No (ref.)				
Yes	1.634 * (0.212)	1.208 * (0.198)	1.588 * (0.218)	1.325 * (0.107)
(9) Paying LTC by welfare				
No (ref.)				
Yes	0.678 * (0.185)	0.854 * (0.127)	0.508 ** (0.185)	0.752 ** (0.111)
(10) Ability to afford premium				
No (ref.)				
Yes	1.474 ** (0.128)	1.008 ** (0.145)	1.775 * (0.113)	1.025 * (0.096)
3.Needs factors				
(11) Likelihood of needing LTC				
No (ref.)				
Yes	1.376 (0.155)	1.765 * (0.128)	1.125 (0.147)	1.557 * (0.101)
(12) Anticipation of being dependent				
No (ref.)				
Yes	1.822 ** (0.209)	2.333 ** (0.213)	1.655 ** (0.211)	2.785 ** (0.189)
Constant	0.023	0.025	0.019	0.027

Note. OR = odds ratio; * *p* < 0.1, ** *p* < 0.05, *** *p* < 0.01. SD value is in parentheses.

## Appendix A

**Table A1:** Simplified questionnaire list.

Serial Number	*Question*
1	How old are you?
2	What is your gender?
3	Are you living with your children now?
4	Are you currently married or unmarried?
5	What is your educational background?
6	Will your family member take care of you if you need long-term care?
7	Do you feel there is an economic burden on your family when you need long-term care?
8	Do you have any extra money at the end of each month for discretionary income?
9	Do you currently have some welfare such as government benefits to pay your long-term care cost?
10	Do you think you can afford to pay for commercial long-term care insurance?
11	What is your evaluation of your opportunities to the LTC need (including home care and formal care such as institutional care) in the future?
12	Will you need help to live due to health problems sometime in the future?
